# MOPCS: next-generation nucleic acid molecular biosensor

**DOI:** 10.1093/nsr/nwac149

**Published:** 2022-07-29

**Authors:** Quan Liu, Shang Chen, Li Wang, Zhaojun Duan, Fuquan Xie, Guojun Zhao, Yunde Hou, Dixian Luo

**Affiliations:** Laboratory Medicine Center, Huazhong University of Science and Technology Union Shenzhen Hospital (Nanshan Hospital), China; Laboratory Medicine Center, Huazhong University of Science and Technology Union Shenzhen Hospital (Nanshan Hospital), China; Guangdong Key Laboratory for Biomedical Measurements and Ultrasound Imaging, School of Biomedical Engineering, Shenzhen University Health Science Center, China; Laboratory Medicine Center, Huazhong University of Science and Technology Union Shenzhen Hospital (Nanshan Hospital), China; Guangdong Key Laboratory for Biomedical Measurements and Ultrasound Imaging, School of Biomedical Engineering, Shenzhen University Health Science Center, China; Department of Laboratory Medicine, Shenzhen Children's Hospital, China; The Sixth Affiliated Hospital of Guangzhou Medical University, Qingyuan City People's Hospital, China; National Institute for Viral Disease Control and Prevention, China Center for Disease Control and Prevention, China; Laboratory Medicine Center, Huazhong University of Science and Technology Union Shenzhen Hospital (Nanshan Hospital), China

**Figure 1.**
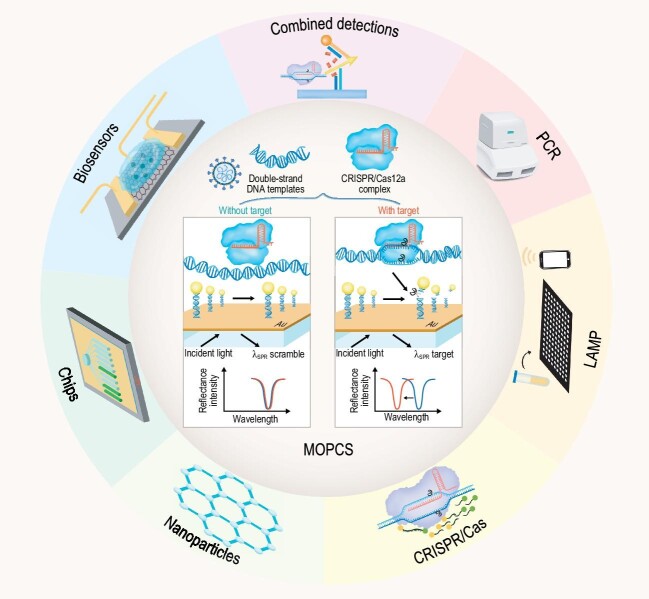
MOPCS as one of major viral nucleic acid detection methods in the future. MOPCS, Methodologies of Photonic CRISPR Sensing.

Severe acute respiratory syndrome coronavirus 2 (SARS-CoV-2) has had a huge impact on the world, causing millions of deaths and a serious blow to the global economy. SARS-CoV-2 can attack most body tissues with devastating consequences [1]. The current global SARS-CoV-2 outbreak has exposed our shortcomings in the prevention and control of infectious diseases, and there is an urgent need for rapid, large-scale and highly efficient diagnostic tools to detect infections in a timely and rapid manner with nucleic acids of virus (Fig. 1). Large-scale and rapid virus screening can block the spread of SARS-CoV-2, protect people's lives and health, and prevent SARS-CoV-2 transmission. Currently, virus screening is still mainly relying on nucleic acid detection based on polymerase chain reaction (PCR) [2]. However, PCR is costly, has low sensitivity, has time-consuming amplification reactions, requires clean environmental conditions and skillful operators, and fails to quickly and efficiently achieve single molecule detection and identification, which hinders large-scale virus screening in most countries. Moreover, novel mutants of SARS-CoV-2 with stronger infectivity continue to appear, but PCR cannot distinguish virus sub-species. Therefore, a novel rapid amplification-free detection method for screening SARS-CoV-2 virus and its variants is urgent and feasible.

A fast, unamplified and accurate testing tool for SARS-CoV-2 diagnosis has become a prerequisite to confirm the exact number of cases worldwide and to take appropriate medical and governmental actions to cut off the virus from spreading. The researchers around the world have been dedicated to developing diverse tests that could output fast and accurate results with high sensitivity and specificity, e.g. developing the Clustered Regularly Interspaced Short Palindromic Repeat (CRISPR)/Cas-based diagnostic principles [3] and facile biosensor technologies [4]. Recently, an interesting article published by Han Zhang *et al*. [5] proposed a promising method based on the CRISPR system and Surface Plasmon Resonance (SPR)-sensing technology, named Methodologies of Photonic CRISPR Sensing (MOPCS), which can detect and distinguish SARS-CoV-2 sub-species without the need for amplification. This system can detect SARS-CoV-2 within 38 min from sample input to results output, and achieve a limit of detection of 15 fM. For the advantage of highly sensitive analysis, MOPCS can detect SARS-CoV-2 and distinguish its variants such as B.1.617.2 (Delta), B.1.1.529 (Omicron) and BA.1 (a subtype of Omicron).

Zhang *et al*. used a highly sensitive optical inspection system, SPR, and designed an Au nanoparticle (AuNP) modified DNA reporter to enhance the SPR signal; therefore, this method achieves a lower detection limit than other CRISPR systems based on the DNA-detection method without amplification, such as fluorescent reporters [5], electrochemical biosensors [6] and some nanobiosensors [7]. This may indicate that SPR is a promising platform for developing an amplification-free device. Furthermore, Zhang *et al*. carried out the application of the single base mutation recognition ability of CRISPR/Cas12a on the SPR platform, highlighting the potential of precise sub-species detection application, which is a novel discovery. In addition, the clinical application potential of MOPCS remains to be further studied and anticipated in more virus-detection fields.

In short, Zhang and co-workers provided an outstanding study in the field of SARS-CoV-2 detection with MOPCS. The results of this study shed light on gene detection research and may replace PCR in large-scale virus screening in the future.
